# Potential factors influencing the academic performance of pharmacy undergraduates: a cross-sectional survey

**DOI:** 10.1186/s12909-026-08984-4

**Published:** 2026-03-14

**Authors:** Abrar F. Alshehri, Abdullah S. Alshammari, Abdulfattah Y. Alhazmi

**Affiliations:** 1 Department of Pharmaceutical Services, College of Pharmacy, Umm Al-Qura University, P.O. Box: 13578, Makkah, 21955 Saudi Arabia; 2 Department of Pharmaceutical Services, Head of Internship Unit, College of Pharmacy, Umm Al-Qura University, P.O. Box: 13578, Makkah, 21955 Saudi Arabia

**Keywords:** Factor, GPA, Pharmacy, Survey, Undergraduate

## Abstract

**Background:**

Academic performance reflects the capability of university students to meet the demands of the pharmacy workforce and professional practice environment. Understanding the factors influencing academic success is essential for improving educational outcomes. This study aims to identify and analyse the factors associated with high grade point averages (GPA) among undergraduate pharmacy students at Umm Al-Qura University (UQU), Saudi Arabia.

**Methods:**

A cross-sectional survey was conducted among pharmacy students enrolled in years 2–6 at the College of Pharmacy, UQU. Data were collected electronically via a self-administered questionnaire distributed through Google Forms. The survey captured demographic, social, economic, transportation, entertainment, motivational, and study-related factors. The Pearson Chi-square test was applied to examine associations between these factors and GPA. A GPA cutoff of ≥ 3.5 (on a 4-point scale) was used to classify students into high and low GPA groups.

**Results:**

A total of 251 students participated in the study (62.5% with GPA < 3.5; 37.5% with GPA ≥ 3.5). The overall response rate was 35.5%. Significant associations were found between high GPA and female gender (*p* < 0.0001), year of study (*p* < 0.0001), lower personal monthly income (*p* = 0.013), and use of private transportation (*p* < 0.0001). High GPA students were also less likely to be responsible for driving family members (*p* = 0.032), spent less time on hobbies (*p* = 0.028), and demonstrated greater motivation to study (*p* = 0.004) and a history of high academic performance (*p* < 0.0001). Additionally, they studied alone more frequently (*p* = 0.028), studied longer daily (*p* = 0.043) and on weekends (*p* = 0.001), and had higher lecture attendance rates (*p* = 0.023). No significant associations were observed with social status, family income, entertainment, or English proficiency.

**Conclusion:**

High academic achievement (GPA ≥ 3.5) among pharmacy students at UQU was significantly linked to gender, study level, personal income, transportation, motivation, study habits, and lecture attendance. These findings highlight modifiable factors that can be targeted to enhance student academic performance.

**Supplementary Information:**

The online version contains supplementary material available at 10.1186/s12909-026-08984-4.

## Introduction

Improving academic performance among pharmacy students has become an international priority. Smith et al. (2007), Alrakaf et al. (2015), Hall et al. (2015), Ubaka et al. (2015), and Okogbaa et al. (2020) have identified key determinants of academic success, including job-related characteristics, mastery-oriented approaches, proficiency in English, employment in non-pharmaceutical fields, and effective time management strategies [1–5]. However, Hall et al. [3], Alqudah et al. [6], and Okogbaa et al. [5] reported that work-avoidance strategies, high workloads, and sleep deprivation are associated with suboptimal academic performance [3, 5, 6]. These mixed findings highlight the need to further investigate how behavioural and demographic characteristics collectively influence academic performance, particularly within specific institutional and cultural settings.

The impact of social and lifestyle choices on academic performance remains uncertain. In the study by Yamamoto and Ishii (2020), approximately half of the pharmacy students surveyed reported using energy drinks before exams, while more than 80% consumed coffee; however, no significant correlation was found between these habits and academic achievement [7]. Similarly, Ghassemi et al. (2019) found no significant association among personality types, social media use, reading proficiency, and academic performance among third-year pharmacy students [8]. Conversely, anxiety, affecting approximately 49% of the student population, has been linked to poor academic performance by Panthee et al. (2010), Ubaka et al. (2015), and Samreen et al. (2020) [4, 9, 10]. These mixed findings underscore the need for further examination of lifestyle and psychosocial variables to clarify their potential role in academic achievement.

Gender differences have also been reported as factors influencing student grade point averages (GPA). Silva and Figueiredo-Braga (2019) and Spivey et al. (2020) found that although female students often experience higher levels of stress, burnout, and emotional fatigue, they tend to achieve higher academic success, demonstrating better time management and more effective study habits than male students [11, 12]. In contrast, Hall et al. (2015), Ubaka et al. (2015), and Spivey et al. (2020) reported that male students were more likely to perform better on the Pharmacy Curriculum Outcomes Assessment [3, 4, 12]. These divergent results suggest that gender may interact with behavioural and academic variables in complex ways, justifying its inclusion in the current analysis.

Al Shawwa et al. (2015) extensively examined factors influencing medical students’ academic achievement, identifying student responsibilities to their families, motivation, English proficiency, study habits, study methods, and vacation habits as significant contributors [13]. These findings provide an empirical rationale for examining both behavioural and demographic factors in relation to academic achievement.

Academic resilience, defined as the ability to overcome academic challenges and stress to achieve positive educational outcomes, is believed to affect pharmacy student performance. Chisholm-Burns et al. (2019) developed a valid and reliable instrument, the Academic Pharmacy Resilience Scale (APRS-16), to assess the influence of academic resilience on the performance of first-, second-, and third-year pharmacy students [14]. However, Chisholm-Burns et al. (2021) reported that the APRS-16 showed no significant association between academic resilience and performance in a first-year mathematics course [15]. This inconsistency highlights a gap in the literature and raises questions about the broader role of academic resilience across different educational contexts.

Despite the growing body of research, there remains limited and inconsistent evidence regarding the demographic, behavioural, lifestyle, and psychological factors that influence academic performance among pharmacy students, and the role of academic resilience remains insufficiently explored. Therefore, the primary aim of this study is to identify and analyse factors associated with GPA (≥ 3.5 vs. < 3.5) among pharmacy students at Umm Al-Qura University in Makkah, Saudi Arabia, during the second semester of 2022. Based on the theoretical and empirical rationale, we hypothesise that demographic factors (e.g., gender, year of study), behavioural factors (e.g., study habits, lecture attendance, motivation), and academic resilience are associated with differences in GPA.

## Methods

This cross-sectional survey was conducted between January and March 2022 at the Faculty of Pharmacy, UQU, Makkah, Saudi Arabia. The study population comprised undergraduate pharmacy students enrolled in the second through sixth academic years during the 2021–2022 academic year. Inclusion criteria were full-time undergraduates enrolled in the Doctor of Pharmacy (PharmD) program who provided informed consent to participate. Excluding first-year students, who may not yet have fully adapted to the curriculum, and graduate or postgraduate students, whose academic performance is influenced by different factors.

### Sample size

Out of 707 eligible undergraduate students enrolled in the PharmD program, a total of 251 students participated in the study, yielding a response rate of 35.5%. The minimum recommended sample size was calculated as 250 students at a 95% confidence level, with a 5% margin of error and an assumed response distribution of 50%, using the Raosoft® online sample size calculator. The achieved sample size therefore met the minimum requirement for adequate statistical power and representativeness of the study population.

### Data collection methods, instruments used, and measurements

Data on potential factors influencing academic performance were collected using a structured, self-administered questionnaire. Academic performance was evaluated based on the cumulative GPA officially obtained from the academic affairs office. The average GPA among students, as obtained from official college reports, was above 3.4 out of 4. Therefore, a GPA cutoff of ≥ 3.5 was used to distinguish students with higher academic achievement from those with lower GPA. This cutoff aligns with the institutional grading standards at UQU, where a GPA of 3.5 or higher is classified as “excellent” academic performance.

Responses were collected through Google Forms (https://2u.pw/pyOxG3Ta), which included an introductory statement outlining the study’s objectives, participant consent, intended use of the data, instructions for completion, confidentiality assurances, the voluntary nature of participation, the right to withdraw at any time, and the estimated completion time. The first section of the questionnaire was adapted from a previously published study [13], with minor modifications (specifically question 23), and validated through a pilot study involving 15 students. Face validity was assessed by the primary author, who reviewed all items for clarity, relevance, and appropriateness, and reliability was evaluated using percent agreement, which demonstrated acceptable consistency among responses. This section comprised 36 items covering various domains: demographic information (e.g., gender, study level, and social status), family factors (e.g., family issues and responsibilities), economic status (e.g., family and student income), transportation (e.g., mode of transport, family transport responsibilities, and car sharing), entertainment (e.g., time spent watching movies, social networking, and hobbies), lifestyle (e.g., smoking and caffeine consumption), social activity (e.g., social hours, extracurricular participation, and conference attendance), sleeping habits (e.g., total sleep duration and nap frequency), motivation (e.g., pursuit of scholarships or university employment), and study-related factors (e.g., group study, English proficiency, study time, study resources, attendance, study methods, study difficulties, and habits). Additional questions addressed vacation and exam habits, as well as students’ academic resilience.

The second section of the questionnaire assessed academic resilience using the validated 16-item *Academic Pharmacy Resilience Scale (APRS-16)* [14], which employed a five-point Likert-type scale ranging from 1 (“Unlikely”) to 5 (“Likely”). Finally, students’ cumulative grade point averages (GPA) were obtained from the academic affairs office to enable comparison of these factors with academic performance.

### Data management and analysis plan

Raw data were collected, cleaned, and recorded in Microsoft Excel before being imported into IBM® SPSS® Statistics version 28 (IBM Corp., Armonk, NY, USA) for analysis. Both descriptive and inferential statistical analyses were conducted. Descriptive statistics summarized participants’ demographic and academic characteristics, as shown in Table 1. The Pearson Chi-square test was first applied to assess associations between categorical variables and GPA categories (high GPA ≥ 3.5 vs. low GPA < 3.5). To satisfy Chi-square assumptions and avoid sparse data, limited recoding was performed. *Social status* (single, married, divorced) was merged into unmarried and married groups, social networking time (“ < 2 h” and “I do not use it”) were grouped together due to few non-users, and all attendance variables originally rated on a five-point scale (100%, 75%, 50%, 25%, I do not attend) were reclassified as > 50% and ≤ 50%. Variables showing significant bivariate relationships were entered into a multiple regression model to identify independent predictors of academic performance. The selected variables were conceptually independent and chosen based on theoretical relevance to ensure meaningful interpretation while controlling for potential confounding factors.

**Table 1:** Baseline characteristics between students’ high vs. low GPA^a^

Baseline characteristics		GPA < 3.5 *N =* 157 (62.5%)	GPA ≥ 3.5 *N =* 94 (37.5%)	Total *N =* 251 (%)	*p*-value
Gender	Male	86 (54.8)	26 (27.7)	112 (44.6)	< 0.0001*
	Female	71 (45.2)	68 (72.3)	139 (55.4)	
Year of study	2nd	11 (7)	39 (41.5)	50 (19.9)	< 0.0001*
	3rd	17 (10.8)	14 (14.9)	31 (12.4)	
	4th	27 (17.2)	9 (9.6)	36 (14.3)	
	5th	42 (26.8)	17 (18.1)	59 (23.5)	
	6th (Internship year)	60 (38.2)	15 (16)	75 (29.9)	
Social status	Unmarried	148 (94.3)	92 (97.9)	240 (95.6)	0.177
	Married	9 (5.7)	2 (2.1)	11 (4.4)	

*N* Number of participants

^a^Grade point averages

^*^Statistically significant

The Kruskal–Wallis H test was used to compare GPA across levels of student and family income, and the Mann–Whitney U test was applied to compare GPA distributions between male and female students. The Kolmogorov–Smirnov test was used to assess data normality. Statistical significance was set at *p* < 0.05 for all tests.

## Results

### Baseline characteristics

A total of 251 PharmD students were included in the analysis, of whom 157 (62.5%) had GPA < 3.5 and 94 (37.5%) had GPA ≥ 3.5. Female students were significantly more likely to have a high GPA than male students (72.3% vs. 27.7%, *p* < 0.0001), and logistic regression showed that females had 3.54 higher odds of achieving a GPA ≥ 3.5 compared with males (95% CI: 2.0–6.2). Although most students were unmarried overall, the proportion of unmarried students with higher GPA (97.9%) was slightly higher than among those with lower GPA (94.3%); however, marital status was not significantly associated with academic performance. The year of study was also significantly related to GPA, with 2nd-year students more frequently achieving high GPA compared with students in later years (*p* < 0.0001) (Table 1).

### Social and family factors

There was no statistically significant difference in GPA between students who lived with their families, those who reported family problems, or those who were socially responsible for caring for a family member (*p* > 0.05). Additionally, the number of family members did not differ significantly between students with higher versus lower GPA (*p* = 0.939) (see Additional file 1).

### Economic factors

GPA was not significantly associated with family income (*p* = 0.489) or with having financial responsibilities for a family member (*p* = 0.378). In contrast, personal monthly income was significantly associated with academic performance. Students earning < Saudi riyal (SAR) 1000 were more likely to achieve a higher GPA compared with those earning ≥ SAR 1000 (*p* = 0.013) (Table 2). A Kruskal–Wallis H test further confirmed significant differences in GPA across income groups, H(2) = 15.497, *p* = 0.001. Post-hoc analysis showed that students with a monthly income < SAR 1000 had the highest mean rank GPA (3.47), followed by those earning SAR 1000–3000 (3.28) and > SAR 3000 (3.26). Students earning < SAR 1000 had significantly higher GPA scores than those earning SAR 1000–3000 (*p* = 0.009) and > SAR 3000 (*p* = 0.017).

**Table 2:** Economic status of students’ high vs. low GPA^a^

Economic status		GPA < 3.5 *N =* 157 (62.5%)	GPA ≥ 3.5 *N =* 94 (37.5%)	Total *N =* 251 (%)	*p*-value
Family’s monthly income	< 5,000 SAR	13 (8.3)	9 (9.6)	22 (8.8)	0.489
	5,000–20,000 SAR	100 (63.7)	65 (69.1)	165 (65.7)	
	> 20,000 SAR	44 (28)	20 (21.3)	64 (25.5)	
Student’s monthly income	< 1,000 SAR	74 (47.1)	61 (64.9)	135 (53.8)	0.013*
	1,000–3,000 SAR	56 (35.7)	26 (27.7)	82 (32.7)	
	> 3,000 SAR	27 (17.2)	7 (7.4)	34 (13.5)	
Financially responsible^b^	No	140 (89.2)	87 (92.6)	227 (90.4)	0.378
	Yes	17 (10.8)	7 (7.4)	24 (9.6)	

*N* Number of participants

^a^Grade point averages

^b^Financially responsible for a family member

^*^Statistically significant; SAR: Saudi riyal

### Transportation factors

The transportation method was significantly associated with GPA (*p* < 0.0001). Students with high GPA more commonly relied on a private driver (39.4% vs. 17.8%), whereas those with lower GPA more frequently drove themselves (57.3% vs. 28.7%). Additionally, being responsible for driving family members was significantly associated with lower GPA (*p* = 0.032) (Table 3).

**Table 3:** Transportation factors of students’ high vs. low GPA^a^

Transportation factors		GPA < 3.5 *N =* 157 (62.5%)	GPA ≥ 3.5 *N =* 94 (37.5%)	Total *N =* 251 (%)	*p*-value
Method of transportation to college	Private driver	28 (17.8)	37 (39.4)	65 (25.9)	< 0.0001*
	With a family member	32 (20.4)	14 (14.9)	46 (18.3)	
	I drive my own car	90 (57.3)	27 (28.7)	117 (46.6)	
	Public transportation (taxi, bus, etc.)	7 (4.5)	16 (17)	23 (9.2)	
Sharing transportation with others	No share	113 (72)	68 (72.3)	181 (72.1)	0.950
	Yes	44 (28)	26 (27.7)	70 (27.9)	
Responsible for driving other family members	No	77 (49)	42 (44.7)	119 (47.4)	0.032*
	Yes	44 (28)	17 (18.1)	61 (24.3)	
	I don’t drive	36 (23)	35 (37.2)	71 (28.3)	

*N* Number of participants

^a^Grade point averages

^*^Statistically significant

### Entertainment factors

Time spent on television, networking, and social activities was not associated with GPA. However, daily engagement in hobbies was more common among students with lower GPA (22.9% vs. 10.6%, *p* = 0.028) (Table 4).

**Table 4:** Entertainment factors of students’ high vs. low GPA^a^

Entertainment factors		GPA < 3.5 *N =* 157 (62.5%)	GPA ≥ 3.5 *N =* 94 (37.5%)	Total *N =* 251 (%)	*p*-value
Hours spent on movies, series, and music/day	< 2 h/day	55 (35.0)	46 (48.9)	101 (40.2)	0.132
	3–4 h/day	67 (42.7)	29 (30.9)	96 (38.2)	
	> 4 h/day	22 (14)	10 (10.6)	32 (12.7)	
	I do not watch television	13 (8.3)	9 (9.6)	22 (8.8)	
Hours spent on networking	< 2 h or I do not use it	33 (21)	21 (22.3)	54 (21.5)	0.239
	3–4 h	56 (35.7)	42 (44.7)	98 (39)	
	> 4 h	68 (43.3)	31 (33.0)	99 (39.4)	
Time spent on hobbies	Every day	36 (22.9)	10 (10.6)	46 (18.3)	0.028*
	Once every week	61 (38.9)	32 (34)	93 (37.1)	
	At least once every month	29 (18.5)	24 (25.5)	53 (21.1)	
	I do not have a hobby	31 (19.7)	28 (29.8)	59 (23.5)	

*N* Number of participants

^a^Grade point averages

^*^Statistically significant

### Motivational factors

Students with high GPA demonstrated stronger intrinsic academic motivation. They were more likely to report enjoying studying (51.1% vs. 32.5%, *p* = 0.004) and to have a lifelong history of high academic achievement (46.8% vs. 15.3%, *p* < 0.0001). Other motivational constructs, including family pressure and scholarship-related motivation, were not significantly different between groups (Table 5).

**Table 5:** Motivational factors of students’ high vs. low GPA^a^

Motivational factors	GPA < 3.5 N (%)	GPA ≥ 3.5 N (%)	*p*-value
I enjoy studying	51 (32.5)	48 (51.1)	0.004*
I have always had high scores	24 (15.3)	44 (46.8)	< 0.0001*
I feel pressured by my family	37 (23.6)	15 (16)	0.15
I want to get a scholarship/be hired by the university	92 (58.6)	44 (46.8)	0.07
I do not feel motivated	41 (26.1)	15 (16)	0.061

*N* Number of participants

^a^Grade point averages

^*^Statistically significant

### Study-related factors

High-performing students exhibited more effective study behaviours. They were more likely to study alone (87.2% vs. 75.2%, *p* = 0.028), study more hours per day (*p* = 0.043), and dedicate more study hours during weekends (*p* = 0.001). Class attendance also differed, with a higher proportion of high-GPA students attending > 50% of lectures (97.9% vs. 90.4%, *p* = 0.023).

In contrast, GPA was not significantly associated with English proficiency—with all teaching, learning, and assessments conducted in English—nor with preferred study resources, study methods, strategies for handling study difficulties, or general study habits (all *p* > 0.05). Similarly, attendance at tutorials, practical sessions, and clinical activities did not differ significantly between the groups (please see Table 6 and Additional file 1).

**Table 6:** Factors affecting the study of students’ high vs. low GPA^a^

Studying factors		GPA < 3.5 *N =* 157 (62.5%)	GPA ≥ 3.5 *N =* 94 (37.5%)	Total *N =* 251 (%)	*p*-value
Group studying	Studying alone	118 (75.2)	82 (87.2)	200 (79.7)	0.028*
	Studying with colleagues	27 (17.2)	11 (11.7)	38 (15.1)	
	Studying in groups	12 (7.6)	1 (1.1)	13 (5.2)	
English proficiency	Fluent English	25 (15.9)	18 (19.1)	43 (17.1)	0.25
	English is good enough to study	119 (75.8)	73 (77.7)	192 (76.5)	
	English is deficient	13 (8.3)	3 (3.2)	16 (6.4)	
Studying hours/day	< 2 h/day	25 (15.9)	16 (17)	41 (16.3)	0.043*
	3–4 h/day	66 (42)	46 (48.9)	112 (44.6)	
	> 4 h/day	19 (12.1)	18 (19.1)	37 (14.7)	
	I do not study daily	47 (29.9)	14 (14.9)	61 (24.3)	
Studying hours at weekends	< 5 h/day	59 (37.6)	41 (43.6)	100 (39.8)	0.001*
	5–8 h/day	41 (26.1)	31 (33.0)	72 (28.7)	
	> 8 h/day	5 (3.2)	10 (10.6)	15 (6)	
	I do not study at weekends	52 (33.1)	12 (12.8)	64 (25.5)	
Lectures attendance	More than 50%	142 (90.4)	92 (97.9)	234 (93.2)	0.023*
	50% and less	15 (9.6)	2 (2.1)	17 (6.8)	
Tutorial attendance	More than 50%	128 (81.5)	78 (83)	206 (82.1)	0.772
	50% and less	29 (18.5)	16 (17)	45 (17.9)	
Practical sessions attendance	More than 50%	125 (79.6)	78 (83)	203 (80.9)	0.512
	50% and less	32 (20.4)	16 (17)	48 (19.1)	
Problem-based learning attendance	More than 50%	88 (56.8)	58 (61.7)	148 (59)	0.444
	50% and less	67 (43.2)	36 (38.3)	103 (41)	
Clinical teaching attendance	More than 50%	88 (56.1)	50 (53.2)	138 (55)	0.659
	50% and less	69 (43.9)	44 (46.8)	113 (45)	
Studying difficulties	Seek clearance independently	34 (21.7)	23 (24.5)	57 (22.7)	0.156
	Ask a colleague	95 (60.5)	47 (50)	142 (56.6)	
	Ask a faculty member	17 (10.8)	19 (20.2)	36 (14.3)	
	Skip it	11 (7)	5 (5.3)	16 (6.4)	

*N* Number of participants

^a^Grade point averages

^*^Statistically significant

Other factors showed no significant associations with GPA. Social status, family status, entertainment activities, lifestyle factors including (smoking, caffeine consumption, sleep duration), social activity, extracurricular involvement, vacation habits, exam preparation habits, and academic resilience did not differ significantly between GPA groups (see Additional file 1).

Multiple regression analysis was conducted using social status, economic status, entertainment factors, social life, sleep habits, study habits, motivation, and academic resilience as predictors of GPA. The model was statistically significant, *F* (7, 237) = 2.848, *p* < 0.01.

## Discussion

Most students fell into the low GPA category, with a nearly equal distribution between males and females, and the majority were in their fifth and sixth years of study. This distribution provides context for interpreting the observed correlations between academic performance and other factors, highlighting potential areas for support and targeted interventions.

### Baseline characteristics

#### Gender & GPA

Gender appears to be an important factor in academic performance, with female students more likely to achieve higher GPA. In this study, females demonstrated competence levels up to three times higher than male students.

Although Al-Shawa et al. (2015) did not report an effect of gender, several studies, including Ali and Naylor (2010), Timer and Clauson (2011), Wan Chik et al. (2012), and Salamonson et al. (2014), support an association between gender and academic achievement, indicating that female students frequently outperform their male counterparts [13, 16–19].

This discrepancy has been explained by Steele and Aronson (1995), Spencer et al. (2016), and Panadero et al. (2017) as potentially arising from differences in study habits, academic motivation, and social or cultural norms and stereotypes [20–22]. Furthermore, Silva et al. (2021) and Haeuser et al. (2022) identify stress perception as a factor contributing to the academic success of female students, which may help explain their higher GPA [23, 24]. These findings suggest that academic performance initiatives may need to focus more on supporting male students.

#### Year of study

Students in the first two years of the pharmacy program had significantly higher GPA compared to those in later years. This finding contrasts with Al-Shawwa et al. (2015), who reported no correlation between academic achievement and year of study [13]. The higher GPA observed in the early years may reflect an adjustment period during the first year, followed by a decline as academic demands increase.

Factors contributing to this decrease, as noted by Ali and Naylor (2010), Klassen et al. (2012), Alshammari et al. (2017), and Ghassemi et al. (2019), may include a more rigorous curriculum and greater involvement in extracurricular activities, although this study did not find a significant association between extracurricular activities and GPA [8, 16, 25, 26]. Academic difficulties and increased workload in the advanced years highlight the need for additional support systems to help students maintain academic performance in advanced years.

### Social and family factors

#### Marital status

The study did not find statistically significant differences in academic achievement between married and unmarried students, despite the limited representation of married students. Most participants were single, and the proportion of single students with lower GPA was slightly higher than those with higher GPA, similar to the findings of Al-Shawwa et al., which focused on medical students [13].

#### Family responsibilities and problems

Consistent with Christenson et al. (1992), Robinson (1993), and Al-Shawwa et al. (2015), there was no significant difference in GPA between students who faced family problems or had responsibilities toward their families. This contrasts with other studies that reported a negative association between family challenges and academic achievement [13, 27, 28]. These findings suggest that, in this study population, family responsibilities and personal challenges do not appear to compromise academic performance, highlighting the resilience and adaptability of the students.

### Economic factors

Students with monthly incomes < 1000 SAR had higher GPA, while those earning > SAR 1000 had lower GPA, suggesting that a lower personal income may motivate students to achieve better academic performance. It should be noted that in our study, “monthly income” refers primarily to government allowances provided to full-time undergraduate students at Umm Al-Qura University rather than wages from employment. Part-time work is uncommon, so most students rely on these stipends, which range from approximately 850 to 1000 SAR per month, depending on the field of study. This finding contrasts with Al-Shawwa et al., who reported that monthly income was not a significant factor (*p* > 0.05) [13]. Another possible explanation is that students with higher personal incomes may spend more time working, taking side jobs, or managing other responsibilities, which could reduce the time available for studying and negatively affect their GPA.

### Transportation factors

Students who used private drivers or public transportation (taxi, bus, etc.) had higher GPA, whereas those who drove their own vehicles had lower GPA. Similarly, students whose families relied on them for transportation tended to perform worse academically, suggesting that added transportation responsibilities may detract from study time and focus. These findings are largely consistent with Al-Shawwa et al., who reported no effect of transportation on GPA, except for the impact of responsibilities related to transporting family members [13]. Importantly, male students who were responsible for driving other family members throughout the day had significantly lower GPAs. Female students, in contrast, generally did not drive and relied on family members or private drivers. Although women in Saudi Arabia have been permitted to drive since 2018, many still depend on others for transportation due to convenience or cultural practices. These findings suggest that transportation responsibilities, particularly driving family members, may reduce study time and academic focus, highlighting a gendered dimension of transportation-related academic impact.

### Entertainment

There was no significant correlation between high GPA and entertainment activities (e.g., watching movies or using social networking sites), consistent with Kirschner and Karpinski (2010) and Bickerdike et al. (2016) [29, 30]. However, some studies, including Anand (2007), Junco (2012), Rosen et al. (2013), and Al-Shawwa et al. (2015), have reported that excessive social media use can negatively impact academic performance [13, 31–33]. Similarly, while Kuśnierz et al. (2020), Spivey et al. (2020), Wu et al. (2020), and Aparisi et al. (2021) found that less involvement in hobbies correlated with higher GPA [12, 34–36], Al-Shawwa et al. reported no significant effect of personal hobbies [13]. These findings suggest that moderate recreational activities, including hobbies and social media use, may not necessarily harm academic performance. Students may benefit more from strategies that focus on effective time management and balancing leisure with academic responsibilities, rather than complete restriction of entertainment activities.

### Motivational factors

Motivational factors were found to significantly influence academic performance. Students who enjoyed studying or consistently received good grades had higher GPA, whereas those motivated primarily by scholarships had lower GPA. This finding is similar to that of Al-Shawwa et al. (2015), which involved medical students [13]. The underlying reasons for lower GPA among scholarship-motivated students are not clear and warrant further investigation in future studies. Furthermore, family-related stress was associated with lower academic performance, consistent with previous studies showing that anxiety and stress impede academic achievement [4, 34, 36, 37]. These findings suggest that enhancing academic motivation may help improve student performance.

### Study-related factors

High-GPA students tended to study independently, dedicating more hours to daily and weekend study, and consistently attending more than 50% of lectures. These findings suggest that promoting independent study, structured study schedules, and regular lecture attendance may help improve academic outcomes. When encountering studying difficulties, students in both GPA groups predominantly relied on peer support rather than consulting faculty members, indicating that help-seeking behaviours were similar regardless of academic performance. This pattern highlights the potential importance of peer-assisted learning while also suggesting opportunities to further encourage appropriate faculty engagement when academic challenges arise.

#### Study duration and frequency

In our study, a positive correlation was observed between study duration and academic performance, with students who studied daily or on weekends achieving higher GPA. In contrast, Al-Shawwa et al. (2015) did not find a significant association between study duration and academic achievement among medical students [13]. This difference may reflect variations in curriculum, teaching methods, student population, or study habits between the two settings, suggesting that study time may play a more critical role in pharmacy education than in the population studied by Al-Shawwa et al.

#### Independent vs. group study

In our cohort, high-GPA students tended to study independently, whereas students with lower GPA were more likely to study with classmates or in groups, consistent with Al-Shawwa et al. [13]. Although group study can foster social interactions, it was sometimes found to distract from academic pursuits, a finding supported by Raychaudhuri et al. [38]. These results suggest that promoting structured independent study habits, while using group study strategically and with clear objectives, may help improve academic outcomes for students.

#### Study techniques and learning strategies

In contrast to Al-Shawwa et al. (2025) English proficiency in our study showed only a non-significant trend toward higher GPA, and factors such as problem-solving skills, specific study techniques, problem-based learning, and focused study environments were not significantly associated with academic performance [13]. The differences may reflect variations in curriculum, teaching methods, or student characteristics between the two GPA groups.

#### Lecture attendance

Al-Shawwa et al. also reported no significant effect of lecture attendance on GPA. However, a significant proportion of high-GPA students in our cohort attended more than 50% of lectures, suggesting that regular lecture attendance is linked to better academic performance.

### Academic resilience

The impact of academic resilience has not been well studied among college or pharmacy students. Trigueros et al. reported that academic resilience positively predicted academic performance among undergraduate students [39], while Popa-Velea et al. (2021) found a significant association between low resilience and poor academic performance in medical students [40]. However, our results showed no effect of higher resilience on GPA, a finding consistent with Chisholm-Burns et al. (2021), who reported that performance in a mathematics course among 374 first-year pharmacy students was not affected by academic resilience [15].

The lack of a significant association between academic resilience and GPA in our cohort may reflect cultural and contextual factors. Students may benefit from strong familial support and deeply held spiritual beliefs, which help them cope with stress rather than directly influence routine academic outcomes, suggesting that environmental and behavioural factors may play a larger role in determining GPA. Further research is needed to explore how resilience interacts with other factors to affect academic outcomes in pharmacy students (see Additional file 1).

### Limitations

Although this study is among the first to broadly examine factors influencing academic performance in pharmacy students, several limitations should be acknowledged. First, the study relied on self-reported data, which may be subject to recall or social desirability bias. Second, it was conducted at a single institution, limiting the generalizability of the findings to other settings. Third, the cross-sectional design allows for the identification of associations but cannot establish causality. Fourth, the study used GPA as a global measure of academic performance, which may not capture the full range of competencies assessed in the pharmacy curriculum. Access to detailed competency-level data at Umm Al-Qura University was not feasible at the time of this study, and future research should incorporate competency-based assessments in line with the Saudi Ministry of Education’s learning outcome criteria. Additionally, the study did not account for all potential confounding variables, such as mental health, learning disabilities, part-time employment, personality traits, or prior academic background, which may influence GPA. Similarly, gender comparisons in this study were based on overall GPA rather than performance in individual courses, so potential variations in gender-related performance across specific subjects could not be assessed.

Moreover, the response rate for this study was 35.5%, which may introduce potential non-response (sampling) bias. Although participants were drawn from all eligible academic years (second to sixth) at UQU, students who chose to respond may differ from non-respondents in motivation, study habits, or academic performance. Therefore, the findings may not fully generalize to the entire population of pharmacy students.

Although multiple regression analyses were conducted to explore the independent associations of predictors with GPA, multicollinearity among predictors was not formally assessed, which may affect the stability of the regression estimates.

Future research should involve larger, more diverse samples across multiple institutions to validate these findings, incorporate competency-based learning outcome measures, and identify effective strategies for enhancing academic achievement.

## Conclusion

This study identified several factors that significantly influence the academic performance of pharmacy students, including gender, year of study, personal economic status, transportation responsibilities, motivation, study habits, lecture attendance and independent study practices. Other factors, such as social status, family responsibilities, entertainment, lifestyle, sleep patterns, hobbies, vacation habits, exam habits, and academic resilience, were not significantly associated with GPA. These findings emphasize the importance of structured, independent study and consistent lecture attendance, and suggest that institutions should offer tailored support systems addressing transportation, financial challenges, and study strategies to enhance student success.

## Supplementary Information

Supplementary Material 1.

Supplementary Material 2.

## Data Availability

Data are available upon request due to privacy and ethical restrictions. The data that support the findings of this study can be requested from the corresponding author, [AFA]. The data are not publicly available because they contain information that could compromise the privacy of research participants.

## Abbreviations

APRS: Academic Pharmacy Resilience Scale
GPA: Grade point averages
UQU: Umm Al-Qura University

## References

[1] Smith L, Saini B, Krass I, Chen T, Bosnic-Anticevich S, Sainsbury E. Pharmacy students' approaches to learning in an Australian university. Am J Pharm Educ. 2007;71(6):120. 10.5688/aj7106120 PMC2690923 19503704

[2] Alrakaf S, Anderson C, Coulman SA, John DN, Tordoff J, Sainsbury E, Rose G, Smith L. An international comparison study of pharmacy students’ achievement goals and their relationship to assessment type and scores. Am J Pharm Educ. 2015;79(3):32. 10.5688/ajpe79335 PMC4428420 25995510

[3] Hall M, Hanna L-A, Hanna A, Hall K: Associations between achievement goal orientations and academic performance among students at a UK pharmacy school. Am J Pharm Educ. 2015;79(5):64. 10.5688/ajpe79564 PMC4571049 26396273

[4] Ubaka CM, Sansgiry SS, Ukwe CV. Cognitive Determinants of Academic Performance in Nigerian Pharmacy Schools. Am J Pharm Educ. 2015;79(7):101.27168614 10.5688/ajpe797101PMC4812777

[5] Okogbaa J, Allen RE, Sarpong DF. Time spent at work and its impact on the academic performance of pharmacy students. Int J Environ Res Public Health. 2020;17(2):496.31941053 10.3390/ijerph17020496PMC7013958

[6] Alqudah M, Balousha SA, Al-Shboul O, Al-Dwairi A, Alfaqih MA, Alzoubi KH. Insomnia among medical and paramedical students in Jordan: impact on academic performance. Biomed Res Int. 2019;2019:7136906. 10.1155/2019/7136906 PMC6875015 31781637

[7] Yamamoto M, Ishii Y. Questionnaire Survey Concerning Pharmacological Cognitive Enhancement among Undergraduates. Yakugaku Zasshi. 2020;140(11):1397–403.33132276 10.1248/yakushi.20-00113

[8] Ghassemi E, Fuller S, Cisneros R, Barnes C, McLendon A, Wilson D. Impact of social media use on reading levels in third-year student pharmacists. Curr Pharm Teach Learn. 2019;11(9):915–9.31570129 10.1016/j.cptl.2019.05.009

[9] Panthee S, Panthee B, Shakya SR, Panthee N, Bhandari DR, Bell JS. Nepalese pharmacy students' perceptions regarding mental disorders and pharmacy education. Am J Pharm Educ. 2010;74(5):89. 10.5688/aj740589 PMC2907854 20798796

[10] Samreen S, Siddiqui NA, Mothana RA: Prevalence of anxiety and associated factors among pharmacy students in Saudi Arabia: a cross-sectional study. Biomed Res Int. 2020;2020:2436538. 10.1155/2020/2436538 PMC7605948 33163532

[11] Silva RG, Figueiredo-Braga M. The roles of empathy, attachment style, and burnout in pharmacy students’ academic satisfaction. Am J Pharm Educ. 2019;83(5):7030. 10.5688/ajpe6706 PMC6630854 31333248

[12] Spivey CA, Chisholm-Burns MA, Johnson JL: Factors associated with student pharmacists’ academic progression and performance on the national licensure examination. Am J Pharm Educ. 2020;84(2):7569 10.5688/ajpe7561 PMC7092787 32226072

[13] Al Shawwa L, Abulaban AA, Abulaban AA, Merdad A, Baghlaf S, Algethami A, et al. Factors potentially influencing academic performance among medical students. Adv Med Educ Pract. 2015;6:65–75.25674033 10.2147/AMEP.S69304PMC4321417

[14] Chisholm-Burns MA, Spivey CA, Sherwin E, Williams J, Phelps S. Development of an instrument to measure academic resilience among pharmacy students. Am J Pharm Educ. 2019;83(6):6896.31507286 10.5688/ajpe6896PMC6718499

[15] Chisholm-Burns MA, Berg-Poppe P, Spivey CA, Karges-Brown J, Pithan A. Resilience and first-year pharmacy students’ academic performance in a pharmacy math course. Am J Pharm Educ. 2021;85(8):8612.34615631 10.5688/ajpe8612PMC8500281

[16] Ali PA, Naylor PB. Association between academic and non-academic variables and academic success of diploma nursing students in Pakistan. Nurse Educ Today. 2010;30(2):157–62.19683845 10.1016/j.nedt.2009.07.006

[17] Wan Chik WZ, Salamonson Y, Everett B, Ramjan LM, Attwood N, Weaver R, et al. Gender difference in academic performance of nursing students in a Malaysian university college. Int Nurs Rev. 2012;59(3):387–93.22897191 10.1111/j.1466-7657.2012.00989.x

[18] Timer JE, Clauson MI. The use of selective admissions tools to predict students’ success in an advanced standing baccalaureate nursing program. Nurse Educ Today. 2011;31(6):601–6.21056921 10.1016/j.nedt.2010.10.015

[19] Salamonson Y, Everett B, Cooper M, Lombardo L, Weaver R, Davidson PM. Nursing as first choice predicts nursing program completion. Nurse Educ Today. 2014;34(1):127–31.23142172 10.1016/j.nedt.2012.10.009

[20] Steele CM, Aronson J. Stereotype threat and the intellectual test performance of African Americans. J Pers Soc Psychol. 1995;69(5):797–811.7473032 10.1037//0022-3514.69.5.797

[21] Spencer SJ, Logel C, Davies PG. Stereotype threat. Annu Rev Psychol. 2016;67:415–37.26361054 10.1146/annurev-psych-073115-103235

[22] Panadero E, Jonsson A, Botella J. Effects of self-assessment on self-regulated learning and self-efficacy: four meta-analyses. Educ Res Rev. 2017;22:74–98.

[23] Silva GO, Aredes NDA, Galdino-Júnior H. Academic performance, adaptation and mental health of nursing students: a cross-sectional study. Nurse Educ Pract. 2021;55:103145.34273732 10.1016/j.nepr.2021.103145

[24] Haeuser E, Serfes AL, Cork MA, Yang M, Abbastabar H, Abhilash ES, et al. Mapping age- and sex-specific HIV prevalence in adults in sub-Saharan Africa, 2000–2018. BMC Med. 2022;20(1):488.36529768 10.1186/s12916-022-02639-zPMC9760541

[25] Klassen RM, Perry NE, Frenzel AC. Teachers’ relatedness with students: an underemphasized component of teachers’ basic psychological needs. J Educ Psychol. 2012;104(1):150.

[26] Alshammari F, Saguban R, Pasay-an E, Altheban A, Al-Shammari L. Factors affecting the academic performance of student nurses: a cross-sectional study. J Nurs Educ Pract. 2017;8(1):60.

[27] Christenson SL, Rounds T, Gorney D. Family factors and student achievement: an avenue to increase students’ success. School Psychol Q. 1992;7(3):178.

[28] Robinson E. The effects of family background on pupils’ academic achievement in Mozambique. Int J Educ Dev. 1993;13(3):289–94.

[29] Kirschner PA, Karpinski AC. Facebook® and academic performance. Comput Human Behav. 2010;26(6):1237–45.

[30] Bickerdike A, O’Deasmhunaigh C, O’Flynn S, O’Tuathaigh C. Learning strategies, study habits and social networking activity of undergraduate medical students. Int J Med Educ. 2016;7:230–6.27424041 10.5116/ijme.576f.d074PMC4958349

[31] Anand V. A study of time management: the correlation between video game usage and academic performance markers. Cyberpsychol Behav. 2007;10(4):552–9.17711364 10.1089/cpb.2007.9991

[32] Rosen LD, Whaling K, Rab S, Carrier LM, Cheever NA. Is Facebook creating “iDisorders”? The link between clinical symptoms of psychiatric disorders and technology use, attitudes and anxiety. Comput Human Behav. 2013;29(3):1243–54.

[33] Junco R. The relationship between frequency of Facebook use, participation in Facebook activities, and student engagement. Comput Educ. 2012;58(1):162–71.

[34] Wu H, Li S, Zheng J, Guo J. Medical students’ motivation and academic performance: the mediating roles of self-efficacy and learning engagement. Med Educ Online. 2020;25(1):1742964.32180537 10.1080/10872981.2020.1742964PMC7144307

[35] Kuśnierz C, Rogowska AM, Pavlova I. Examining gender differences, personality traits, academic performance, and motivation in Ukrainian and polish students of physical education: a cross-cultural study. Int J Environ Res Public Health. 2020;17(16):5729. 10.3390/ijerph17165729 PMC7459791 32784806

[36] Aparisi D, Delgado B, Bo RM, Martínez-Monteagudo MC. Relationship between cyberbullying, motivation and learning strategies, academic performance, and the ability to adapt to university. Int J Environ Res Public Health. 2021;18(20):10646. 10.3390/ijerph182010646 PMC8535497 34682391

[37] Balogun AG, Balogun SK, Onyencho CV. Test anxiety and academic performance among undergraduates: the moderating role of achievement motivation. Span J Psychol. 2017;20:E14.28190417 10.1017/sjp.2017.5

[38] Raychaudhuri A, Debnath M, Sen S, Majumder BG. Factors affecting students' academic performance: a case study in Agartala Municipal Council Area. Bangladesh e-J Sociol. 2010;7(2):34–41.

[39] Trigueros R, Padilla A, Aguilar-Parra JM, Mercader I, López-Liria R, Rocamora P. The influence of transformational teacher leadership on academic motivation and resilience, burnout and academic performance. Int J Environ Res Public Health. 2020;17(20):7687. 10.3390/ijerph17207687 PMC7589400 33096777

[40] Popa-Velea O, Pîrvan I, Diaconescu LV. The impact of self-efficacy, optimism, resilience and perceived stress on academic performance and its subjective evaluation: a cross-sectional study. Int J Environ Res Public Health. 2021;18(17):8911. 10.3390/ijerph18178911 PMC8431330 34501501

