# Supplementary material for: Potential factors influencing the academic performance of pharmacy undergraduates: a cross-sectional survey
Source: BMC Med Educ. 2026 Mar 14;26:651. doi: 10.1186/s12909-026-08984-4 (PMC13101140; doi:10.1186/s12909-026-08984-4)
Supplement: Supplementary file 1 — Supplementary Material 1. [file 12909_2026_8984_MOESM1_ESM.pdf]

Other Factors Affecting Students' High & Low GPAs[a]

Affecting factors	GPAs[a] < 3.5		GPAs[a] ≥ 3.5		*p*-value
	N	(%)	N	(%)	
Family status					
Number of family members					
1 to 10 members	150	95.5%	90	95.7%	0.939
11 to 20 members	7	4.5%	4	4.3%	
Living with family					
No	5	3.2%	5	5.3%	0.403
Yes	152	96.8%	89	94.7%	
Family issues					
Divorced parents	8	5.1%	5	5.3%	0.082
Death of a first relative	18	11.5%	16	17.0%	
A family member with chronic illnesses	47	29.9%	15	16.0%	
I do not have any problem	84	53.5%	58	61.7%	
Socially responsible[b]					
No	92	58.6%	60	63.8%	0.412
Yes	65	41.4%	34	36.2%	
Lifestyle					
Smoking					
Smoker	32	20.4%	13	13.8%	0.19
Non-Smoker	125	79.6%	81	86.2%	
Caffeine consumption[c]					
Once every day	76	48.4%	44	46.8%	0.533
Several times every day	44	28.0%	32	34.0%	
I do not consume caffeinated drinks	37	23.6%	18	19.1%	
Social activity					
Spend time with friends					
Every day	26	16.6%	12	12.8%	0.135
<3 times/week	92	58.6%	47	50.0%	
≥ 3 times/week	18	11.5%	12	12.8%	

Do not spend time with my friends	21	13.4%	23	24.5%	
Extracurricular activities[d]					
Every day	11	7.0%	10	10.6%	0.779
Spend <3 times/week with my friends	14	8.9%	8	8.5%	
Spend ≥3 times/week with my friends	53	33.8%	29	30.9%	
Do not spend time on such activities	79	50.3%	47	50.0%	
Medical conferences and courses					
Attend less than four events/year	65	41.4%	39	41.5%	0.201
Attend more than four/year	14	8.9%	3	3.2%	
Sleeping					
Nap habits					
Take a nap before studying, and sleep at night	75	47.8%	46	48.9%	0.657
Do not take naps, and just sleep at night	46	29.3%	23	24.5%	
I am an evening person	36	22.9%	25	26.6%	
Sleeping hours/day					
I sleep <6 hours/day	42	26.8%	32	34.0%	0.43
I sleep 6–8 hours/day	94	59.9%	49	52.1%	
I sleep >8 hours/day	21	13.4%	13	13.8%	
Exam habits					
Preparing for the exam 1 month before exam	74	47.1%	37	39.4%	0.381
Preparing for the exam 2–3 weeks before exam	68	43.3%	44	46.8%	
Preparing for the exam Few days before exam	15	9.6%	13	13.8%	

N: number of participants; [a] Grade point averages; [b] Socially responsible for taking care of anyone in the family other than yourself (e.g.: doing house chores, sick family member, rising a child), [c] Caffeine consumption (coffee, Cola, Red Bull, etc); [d] Extracurricular activities (volunteer and charity work, organizing committees, etc)

Vacation Habits Affecting Students' High & Low GPAs[a]

Vacation habits	GPAs[a] < 3.5		GPAs[a] s ≥ 3.5		*p*-value
	N	(%)	N	(%)	
Start reading for the next year subjects	18	11.5%	11	11.7%	0.955
Have clinical training	35	22.3%	19	20.2%	0.698
Conduct research	17	10.8%	11	11.7%	0.831

Enjoy my vacation	148	94.3%	86	91.5%	0.397

N: number of participants; [a] Grade point averages

Study Resources Affecting Students' High & Low GPAs[a]

Study Resource	Importance Level	GPA < 3.5 N (%)	GPA ≥ 3.5 N (%)	*p*-value
Internet	Most important	67 (42.7%)	42 (44.7%)	0.298
	Very important	23 (14.6%)	22 (23.4%)	
	Important	40 (25.5%)	19 (20.2%)	
	Somewhat important	19 (12.1%)	9 (9.6%)	
	Least important	8 (5.1%)	2 (2.1%)	
Books	Most important	5 (3.2%)	3 (3.2%)	0.999
	Very important	25 (15.9%)	14 (14.9%)	
	Important	30 (19.1%)	18 (19.1%)	
	Somewhat important	33 (21.0%)	19 (20.2%)	
	Least important	64 (40.8%)	40 (42.6%)	
Handouts (Lecture Notes)	Most important	61 (38.9%)	37 (39.4%)	0.586
	Very important	42 (26.8%)	21 (22.3%)	
	Important	35 (22.3%)	22 (23.4%)	
	Somewhat important	9 (5.7%)	10 (10.6%)	
	Least important	10 (6.4%)	4 (4.3%)	
My Own Notes	Most important	12 (7.6%)	11 (11.7%)	0.533
	Very important	54 (34.4%)	29 (30.9%)	
	Important	35 (22.3%)	22 (23.4%)	
	Somewhat important	47 (29.9%)	23 (24.5%)	
	Least important	9 (5.7%)	9 (9.6%)	
Other (Videos)	Most important	12 (7.6%)	1 (1.1%)	0.228
	Very important	13 (8.3%)	8 (8.5%)	
	Important	17 (10.8%)	13 (13.8%)	
	Somewhat important	49 (31.2%)	33 (35.1%)	

	Least important	66 (42.0%)	39 (41.5%)	

N: number of participants; [a] Grade point averages

Study Methods and Habits Affecting Students' High & Low GPAs[a]

Studying factor	GPAs[a] < 3.5<br>N (%)	GPAs[a] ≥3.5<br>N (%)	*p*-value
Study method			
Mapping	42 (26.8)	22 (23.4)	0.556
Forming note	67 (42.7)	48 (51.1)	0.197
Highlighting	93 (59.2)	64 (68.1)	0.161
Summarizing	91 (58)	49 (52.1)	0.368
Recording	63 (40.1)	33 (35.1)	0.428
Reading loudly	73 (46.5)	49 (52.1)	0.388
Reading silently	18 (11.5)	13 (13.8)	0.582
Start by reading then memorizing	96 (61.1)	58 (61.7)	0.930
Start by memorizing	15 (9.6)	11 (11.7)	0.589
Study habits			
Drink coffee	96 (61.1)	67 (71.3)	0.104
Eat snacks	57 (36.3)	31 (33)	0.593
ensure silence and no interruptions	0 (0)	0 (0)	NA
Study in my living room	91 (58)	56 (59.6)	0.802
Favor a certain body position	73 (46.5)	45 (47.9)	0.833
Listening to Qur'an/music and television	11 (7)	10 (10.6)	0.315

N: number of participants; [a] Grade point averages; NA: not applicable; [b] Favor a certain body position (lying on the floor, sitting on a desk).

Academic Resilience Affecting Students' High & Low GPAs[a]

Academic resilience	GPAs[a] < 3.5		GPAs[a] ≥3.5		*p*-value
	N	%	N	%	
I would begin to doubt my chances of success in the PharmD program.					

Unlikely	39	63.9%	22	36.1%	0.265
Somewhat unlikely	19	54.3%	16	45.7%	
Neutral	46	66.7%	23	33.3%	
Somewhat likely	32	71.1%	13	28.9%	
Likely	21	51.2%	20	48.8%	
I would probably get depressed.					
Unlikely	35	60.3%	23	39.7%	0.922
Somewhat unlikely	19	57.6%	14	42.4%	
Neutral	31	63.3%	18	36.7%	
Somewhat likely	40	66.7%	20	33.3%	
Likely	32	62.7%	19	37.3%	
I would be very disappointed.					
Unlikely	37	58.7%	26	41.3%	0.092
Somewhat unlikely	19	47.5%	21	52.5%	
Neutral	44	66.7%	22	33.3%	
Somewhat likely	34	75.6%	11	24.4%	
Likely	23	62.2%	14	37.8%	
I would begin to think my chances of getting the job or residency I want were poor.					
Unlikely	30	58.8%	21	41.2%	0.596
Somewhat unlikely	25	59.5%	17	40.5%	
Neutral	33	62.3%	20	37.7%	
Somewhat likely	46	70.8%	19	29.2%	
Likely	23	57.5%	17	42.5%	
I would feel like everything was ruined and going wrong.					
Unlikely	53	58.9%	37	41.1%	0.863
Somewhat unlikely	24	64.9%	13	35.1%	
Neutral	33	67.3%	16	32.7%	
Somewhat likely	26	60.5%	17	39.5%	
Likely	21	65.6%	11	34.4%	
I would try to think of new solutions					

Unlikely	8	72.7%	3	27.3%	0.355
Somewhat unlikely	8	80.0%	2	20.0%	
Neutral	23	71.9%	9	28.1%	
Somewhat likely	32	55.2%	26	44.8%	
Likely	86	61.4%	54	38.6%	
I would use my past successes to help motivate myself.					
Unlikely	3	50.0%	3	50.0%	0.268
Somewhat unlikely	4	57.1%	3	42.9%	
Neutral	17	77.3%	5	22.7%	
Somewhat likely	33	71.7%	13	28.3%	
Likely	100	58.8%	70	41.2%	
I would set my own goals for achievements.					
Unlikely	3	42.9%	4	57.1%	0.392
Somewhat unlikely	6	85.7%	1	14.3%	
Neutral	15	68.2%	7	31.8%	
Somewhat likely	46	66.7%	23	33.3%	
Likely	87	59.6%	59	40.4%	
I would seek encouragement from my family and friends					
Unlikely	16	57.1%	12	42.9%	0.884
Somewhat unlikely	12	63.2%	7	36.8%	
Neutral	27	61.4%	17	38.6%	
Somewhat likely	42	67.7%	20	32.3%	
Likely	60	61.2%	38	38.8%	
I would try to think about my strengths and weaknesses to help me work better					
Unlikely	1	20.0%	4	80.0%	0.259
Somewhat unlikely	3	60.0%	2	40.0%	
Neutral	14	58.3%	10	41.7%	
Somewhat likely	41	69.5%	18	30.5%	
Likely	98	62.0%	60	38.0%	
I would see the situation as a challenge					

Unlikely	5	62.5%	3	37.5%	0.283
Somewhat unlikely	5	35.7%	9	64.3%	
Neutral	24	64.9%	13	35.1%	
Somewhat likely	42	60.9%	27	39.1%	
Likely	81	65.9%	42	34.1%	
I would do my best to stop thinking negative thoughts.					
Unlikely	4	66.7%	2	33.3%	0.691
Somewhat unlikely	8	80.0%	2	20.0%	
Neutral	24	68.6%	11	31.4%	
Somewhat likely	35	60.3%	23	39.7%	
Likely	86	60.6%	56	39.4%	
I would see the situation as temporary					
Unlikely	9	52.9%	8	47.1%	0.673
Somewhat unlikely	10	55.6%	8	44.4%	
Neutral	40	58.8%	28	41.2%	
Somewhat likely	45	67.2%	22	32.8%	
Likely	53	65.4%	28	34.6%	
I would change my career plans					
Unlikely	22	73.3%	8	26.7%	0.380
Somewhat unlikely	18	69.2%	8	30.8%	
Neutral	39	55.7%	31	44.3%	
Somewhat likely	43	66.2%	22	33.8%	
Likely	35	58.3%	25	41.7%	
I would not change my long-term goals and ambitions					
Unlikely	9	60.0%	6	40.0%	0.174
Somewhat unlikely	9	40.9%	13	59.1%	
Neutral	35	59.3%	24	40.7%	
Somewhat likely	42	64.6%	23	35.4%	
Likely	62	68.9%	28	31.1%	

N: number of participants; [a] Grade point averages
