# Supplementary material for: Potential factors influencing the academic performance of pharmacy undergraduates: a cross-sectional survey
Source: BMC Med Educ. 2026 Mar 14;26:651. doi: 10.1186/s12909-026-08984-4 (PMC13101140; doi:10.1186/s12909-026-08984-4)
Supplement: Supplementary file 2 — Supplementary Material 2. [file 12909_2026_8984_MOESM2_ESM.pdf]

Questionnaire for pharmacy students

1. Sex: □ Male □ Female
2. University number: ………….
3. Year of study: a) 2nd b) 3rd c) 4th d) 5th e) 6th (internship year)
4. Social status:
   a) Single
   b) Married
   c) Divorced
5. Total family number: …………..
6. Do you live with your family: a) Yes b) No
7. Did you have any family problems?
   a) Divorced parents
   b) Death of a first relative during your medical studying
   c) A family member with chronic illnesses
   d) I do not have any problem or death in my family
8. Monthly income of the family:
   a) <5,000 SR
   b) 5,000–20,000 SR
   c) >20,000 SR
9. Your monthly income:
   a) <1,000 SR
   b) 1,000–3,000 SR
   c) >3,000 SR
10. Are you financially responsible for anyone in the family other than yourself?
    a) Yes b) No
11. Modes of transportation to college:
    a) Private driver.
    b) With a family member.
    c) I drive my own car.
    d) Public transportation (taxis, bus).
12. Do you have to share your transportation method with others:
    a) Yes b) No
13. Are you responsible to drive other family members throughout the day?
    a) Yes b) No c) I do not drive.
14. Are you socially responsible for taking care of anyone in the family other than yourself (e.g.: doing house chores, sick family member, rising a child)?
    a) Yes b) No
15. How many hours do you spend on movies, series, and music/day?
    a) <2 hours/day
    b) 3–4 hours/day
    c) >4 hours/day
    d) I do not watch television.

16. How many hours do you spend on social networking/chatting (online/BB) *“not related to college work”*?

a) <2 hours

b) 3–4 hours

c) >4 hours

d) I do not use it.

17. Which of the following sentences is most accurate:

a) I spend some of my time on my hobbies every day.

b) I spend some of my time on my hobbies once every week.

c) I spend some of my time on my hobbies at least once every month.

d) I do not have a hobby.

18. Regarding smoking (cigarettes, cigars, Ma’asel, etc) habits:

a) I am a smoker.

b) I smoke at least once every week.

c) I am a social smoker

d) I do not smoke.

e) I used to smoke but I quit.

19. Regarding consumption of caffeine containing beverages (coffee, Cola, Red Bull, etc), I:

a) Regularly consume such beverages once every day.

b) Regularly consume such beverages several times every day.

c) I do not consume caffeinated drinks.

20. Regarding my social life (outside the university, only for the purpose of socializing), I:

a) Spend time with my friends every day.

b) Spend ≥3 times/week with my friends.

c) Spend <3 times/week with my friends.

d) Do not spend time with my friends.

21. Regarding extracurricular activities (volunteer and charity work, organizing committees, etc), I:

a) Spend at least 5 hours/week on such activities.

b) Spend less than 5 hours/week.

c) Participate in 1–2 events every year.

d) Do not spend time on extracurricular activities.

22. Regarding medical conferences and courses *‘‘not related to college work”*, I:

a) Attend less than four events/year.

b) Attend more than four/year.

c) I do not attend such events.

23. After university hours, I:

a) Take a nap before studying, and sleep at night.

b) Do not take naps, and just sleep at night.

b) I am a *‘‘night owl’’* or evening person *‘‘a person who tends to stay up until late at night, or the early hours of the morning’’*

24. Regarding my sleeping hours/day: (including nap hours)

a) I sleep <6 hours/day.

b) I sleep 6–8 hours/day.

c) I sleep >8 hours/day.

25. I am motivated to study harder because: (you can choose more than one)

a) I enjoy studying.

b) I have always had high scores.

c) I feel pressured by my family.

d) I want to get a scholarship/get hired by the university.

e) I do not feel motivated.

26. When I study:

a) I like to study alone.

b) I like to study with one of my colleagues.

c) I like to study in groups.

27. Regarding my English proficiency:

a) I speak fluent English.

b) My English is good enough to study and understand day-to-day conversations.

c) My English is deficient, that I face some difficulties when I study.

28. During the day, I study:

a) <2 hours/day.

b) 3–4 hours/day.

c) >4 hours/day.

d) I do not study daily.

29. During the weekends, I study:

a) <5 hours/day.

b) 5–8 hours/day.

c) >8 hours/day.

d) I do not study during weekends.

30. Arrange the following resources according to their importance in your studying: from1 to 5, in which 1 is most important and 5 is least important:

a) Internet b) Books c) Handouts d) My own notes e) Other (videos)

31. When I study I use the following: (choose the most important three)

a) Mapping

b) Note forming

c) Highlighting

d) Summarizing

e) Recording

f) Reading loudly

g) Reading silently

h) Start by reading then memorizing

i) Start by memorizing

32. Rate the following according to your attendance

1) 100% 2) 75% 3) 50% 4) 25% 5) I do not attend

a) Lectures

b) Tutorials

c) Practical sessions

d) Problem-based learning

e) Clinical teaching

33. When facing difficulties during studying, I:

a) Seek clearance independently (self-directed learning).

b) Ask a colleague.

c) Ask a faculty member.

d) Skip it.

34. When I study, I: (you can choose more than one)

a) Drink coffee.

b) Eat snacks.

c) Have to ensure silence and no interruptions.

d) Favor a certain place (eg, I only like to study in my living room).

e) Favor a certain body position (lying on the floor, sitting on a desk).

f) Listening to Qur’an/music and television.

35. During vacations, I prefer to: (You can choose more than one)

a) Start reading for the next year subjects.

b) Have clinical training.

c) Conduct research.

d) Enjoy my vacation.

36. In a course of 2–3 months duration, I start preparing for the exam:

a) 1 month before exam.

b) 2–3 weeks before exam.

c) Few days before exam.

Academic Pharmacy Resilience Scale (APRS-16)

	**5= Likely**	**4= Somewhat likely**	**3= Neutral**	**2= Somewhat unlikely**	**1= Unlikely**
1. I would begin to doubt my chances of success in the PharmD program.					
2. I would probably get depressed.					
3. I would be very disappointed.					
4. I would begin to think my chances of getting the job or residency I want were poor.					
5. I would feel like everything was ruined and going wrong.					
6. I would try to think of new solutions.					
7. I would use my past successes to help motivate myself.					
8. I would set my own goals for achievements.					
9. I would seek encouragement from my family and friends.					
10. I would try to think about my strengths and weaknesses to help me work better.					
11. I would see the situation as a challenge.					

12. I would do my best to stop thinking negative thoughts.					
13. I would see the situation as temporary.					
15. I would change my career plans.					
16. I would not change my long-term goals and ambitions.					
